# Microscopic Fractional Anisotropy Detects Cognitive Training-Induced Microstructural Brain Changes

**DOI:** 10.3390/tomography8010004

**Published:** 2022-01-01

**Authors:** Xinnan Li, Daisuke Sawamura, Hiroyuki Hamaguchi, Yuta Urushibata, Thorsten Feiweier, Keita Ogawa, Khin Khin Tha

**Affiliations:** 1 Laboratory for Biomarker Imaging Science, Hokkaido University Graduate School of Biomedical Science and Engineering, Sapporo 060-8638, Japan; LXN@pop.med.hokudai.ac.jp (X.L.); hhummer@huhp.hokudai.ac.jp (H.H.); 2 Department of Rehabilitation Science, Hokkaido University Faculty of Health Sciences, Sapporo 060-0812, Japan; D.sawamura@pop.med.hokudai.ac.jp; 3 Siemens Healthcare K.K., Tokyo 141-8644, Japan; yuta.urushibata@siemens-healthineers.com; 4 Siemens Healthcare GmbH, 91052 Erlangen, Germany; thorsten.feiweier@siemens-healthineers.com; 5 Department of Rehabilitation, Hokkaido University Hospital, Sapporo 060-8648, Japan; ot-ogawa@huhp.hokudai.ac.jp; 6 Global Center for Biomedical Science and Engineering, Hokkaido University Faculty of Medicine, Sapporo 060-8638, Japan

**Keywords:** double diffusion encoding, microscopic fractional anisotropy, microstructure, cognitive training

## Abstract

Cognitive training-induced neuroplastic brain changes have been reported. This prospective study evaluated whether microscopic fractional anisotropy (μFA) derived from double diffusion encoding (DDE) MRI could detect brain changes following a 4 week cognitive training. Twenty-nine healthy volunteers were recruited and randomly assigned into the training (*n* = 21) and control (*n* = 8) groups. Both groups underwent brain MRI including DDE MRI and 3D-T1-weighted imaging twice at an interval of 4–6 weeks, during which the former underwent the training. The training consisted of hour-long dual N-back and attention network tasks conducted five days per week. Training and time-related changes of DDE MRI indices (μFA, fractional anisotropy (FA), and mean diffusivity (MD)) and the gray and white matter volume were evaluated using mixed-design analysis of variance. In addition, any significant imaging indices were tested for correlation with cognitive training-induced task performance changes, using partial correlation analyses. μFA in the left middle frontal gyrus decreased upon the training (53 voxels, uncorrected *p* < 0.001), which correlated moderately with response time changes in the orienting component of attention (r = −0.521, uncorrected *p* = 0.032). No significant training and time-related changes were observed for other imaging indices. Thus, μFA can become a sensitive index to detect cognitive training-induced neuroplastic changes.

## 1. Introduction

Cognitive training is often conducted in normal and cognitively impaired patients to improve cognitive performance [1,2]. Studies that evaluate the training duration have shown that improved cognitive performance can be expected with three weeks or longer training duration [3]. How training modulates the brain structure and function has been a subject of study. While techniques such as single-neuron recording can directly measure the electrophysiological responses of a single neuron with superb temporal and spatial resolution, their applicability is limited due to the invasive nature and small brain coverage [4,5]. Instead, advanced magnetic resonance imaging (MRI) techniques are widely applied to study the brain’s neuroplastic changes following cognitive training, thanks to their capability of showing the brain changes noninvasively and in vivo [1,2,3].

Diffusion tensor imaging (DTI) has often been applied to illustrate the microstructural brain changes associated with improved cognitive performance following the training. Changes in its indices, such as fractional anisotropy (FA) and mean diffusivity (MD), have been reported. For example, a 4 week cognitive training of working memory, attention, and inhibition control resulted in an FA increase in the left superior longitudinal fasciculus in normal adults [6]. An 8 week cognitive training reported an FA increase in the right precentral gyrus and an MD decrease in the left superior longitudinal fasciculus in traumatic brain injury (TBI) patients [2]. These DTI changes have also been shown to associate with efficient decision-making or improved information processing speed and verbal working memory, suggesting this technique’s suitability in revealing cognitive training-induced neuroplastic changes [2,6]. Animal experiments have further documented the applicability of DTI in depicting neural changes at the microstructural level. Studies on rats have shown that learning and memory training-induced FA increase in the corpus callosum and MD decrease in the hippocampus are associated with the local increase in synapses, astrocyte volume, its perimeter and organization, and axon or myelin packing [7].

Like other diffusion imaging schemes, DTI measures the direction and magnitude of motion of water molecules within voxels. However, although DTI can illustrate the microstructural brain changes and has been applied in various physiological and pathological states, it cannot resolve anisotropic structures aligned in several orientations [8,9,10]. A potential solution to this limitation is the application of multiple diffusion encoding (MDE) schemes. Recent improvements in gradient systems of clinical scanners have allowed the acquisition of MDE schemes. An example is double diffusion encoding (DDE) MRI, which applies two diffusion gradient pairs in the parallel and orthogonal directions, with a separation of less than 100 ms time interval. It has been shown that the introduction of a second gradient pair with a variable encoding direction improves the sensitivity in acquiring microstructural information [11,12]. In addition, microscopic fractional anisotropy (μFA), a metric extractable with DDE MRI is believed as capable of quantifying water diffusion anisotropy without the conflating effects of orientation dispersion [12].

Initial studies on DDE MRI have shown the superiority of μFA over FA of DTI in characterizing anisotropic structures. Liquid crystal/yeast and pureed asparagus phantom studies have shown higher resolution in terms of orientation dispersion with μFA than with FA [10]. The superiority of μFA over FA has been reported in detecting age-related white matter changes and white matter lesions in patients with multiple sclerosis and Parkinson’s disease [13,14,15]. Meningiomas and glioblastomas also exhibit different μFA characteristics (i.e., the former have high μFA, whereas the latter have low μFA values), although these two tumor types are indistinguishable by FA [16].

Considering the superiority of μFA in evaluating anisotropic structures, DDE MRI may provide more accurate information about the neuroplastic brain changes following cognitive training. However, to our knowledge, there have been no reports that evaluate cognitive training-induced brain changes by using DDE MRI. Therefore, this study aimed to evaluate if μFA derived from DDE MRI could detect cognitive training-induced neuroplastic changes in healthy young adults, following 4 week attention and working memory training. As a reference, FA and MD derived from DTI were also evaluated.

## 2. Materials and Methods

### 2.1. Participants

All participants were recruited over 16 months (July 2018 through October 2019). The inclusion criterion was age over 20 years. The exclusion criteria were absolute contraindications for MRI, diseases that might affect the integrity of the central nervous system, and previous experience with cognitive training [17]. Participants were divided into training and control groups to evaluate cognitive training-induced neuroplastic changes and the relationship between these changes and the training performance. The sample size needed for this study was estimated using G power 3.0 [18], according to a prospective internal pilot study. To observe cognitive training-induced DDE MRI changes (effect size f = 0.530) and achieve the statistical power of 0.90, a sample size of *n* = 6 would be necessary in each group. To observe a significant correlation between the training-induced changes in DDE MRI indices and cognitive improvement (effect size |r| = 0.611) and achieve a statistical power of 0.90, a sample size of *n* = 16 would be necessary. Considering drop-out, 23 subjects were recruited for the training group and 8 age, sex, and education level-matched subjects for the control group. Of 23 subjects, 2 were excluded due to incomplete cognitive training. Therefore, a total of 21 participants (9 female and 12 male; age range = 22–40 years; mean age ± standard deviation (SD) = 27.7 ± 6.0 years; mean years of education ± SD = 15.7 ± 0.5 years) were included in the training group, and 8 participants (4 female and 4 male; age range = 22–52 years; mean age ± SD = 31.5 ± 9.5 years; mean years of education ± SD = 15.5 ± 1.3 years) were included in the control group.

### 2.2. Procedures

All participants underwent brain MRI twice at an interval of 4 to 6 weeks, during which the training group underwent a 4 week cognitive training of spatial attention and working memory. The former was conducted as a 30 min attention network training (ANT) each day for five days/week, and the latter as another 30 min training of dual N-back training (DBT) each day for five days/week. The performance of each task was assessed on the day of MRI (i.e., before and after the training) to measure the training-induced cognitive improvement.

### 2.3. Cognitive Training

Cognitive training was conducted using a notebook PC (resolution 1366 × 768 pixels, refresh rate 60 Hz).

#### 2.3.1. ANT

The ANT is an attention network task [19]. In this study, DMDX software (University of Arizona, Tuscon, AZ, USA) was used to perform the task [20]. First, a white fixation cross was presented at the screen center. After a time interval of 400 ms, 800 ms, 1200 ms, or 1600 ms (note: this interval was randomly chosen to avoid cue stimulus prediction), a white circular cue stimulus was presented for 100 ms under one of the following three conditions: no-cue (no appearance of cue), center-cue (the appearance of a cue in the same position as the fixation cross), and spatial-cue (the appearance of cue above or below the fixation cross). After a 400 ms interstimulus interval, five white arrows were presented above or below the fixation cross. The center arrow served as the target stimulus and the other as flanker stimuli. The flanker stimuli were presented in either of two conditions: congruent (flanker stimuli in the same direction as the target stimulus) or incongruent (flanker stimuli in the opposite direction to the target stimulus). The target stimulus was presented for 1700 ms. The participant was asked to indicate the direction of the target stimulus by pressing the cursor button “→” or “←” on the keyboard (i.e., the cursor pointing the same direction as the target stimulus) as fast as possible. The ANT task performance was evaluated on the basis of the response time (RT), which is the time taken for the participant to respond by pressing the cursor button after the appearance of the target stimulus. The task performance of each subcomponent network was calculated as RT_alerting_ = RT_no-cue_ − RT_center-cue_, RT_orienting_ = RT_center-cue_ − RT_spatial-cue_, and RT_executive control_ = RT_incongruent_ − RT_congruent_. The RT longer than 1700 ms was considered as an incorrect response. In this study, the changes of RT in alerting, orienting, and executive control between the initial and re-assessment were recorded to measure the ANT-induced cognitive improvement.

#### 2.3.2. DBT

The N-back task involves simultaneous visuospatial and auditory tasks [21]. In this study, the DBT was built using an in-house JAVA 8 script. The visuospatial task was conducted using a white 3 × 3 square presented on the screen. Of the nine squares, a square was highlighted randomly as the visuospatial stimulus. An auditory stimulus that was one of the Japanese phonetic characters, “a”, “i”, “u”, “e”, and “o”, was simultaneously presented. The visuospatial and auditory stimuli were presented for 500 ms and were repeated after a 2500 ms interstimulus interval. In this task, the participant needed to remember the presented visuospatial and auditory stimuli and determine whether the current stimuli (both visuospatial and auditory) were the same as those presented N times before. The level of task difficulty (the value of N) was set to alter automatically. The training session started with a dual 1-back. The difficulty level increased by one number (e.g., dual 2-back) if the participant scored 85% in both tasks for nine consecutive runs. Altogether, 600 stimuli were presented in 30 min. The error rate, which is the ratio of the number of erroneous responses (i.e., erroneous responses for either stimulus or both) to the total number of responses to stimuli, was used to evaluate task performance. The change in error rate between the initial and re-assessment was assessed to measure the DBT-induced cognitive improvement.

### 2.4. MR Imaging

**Figure 1:**
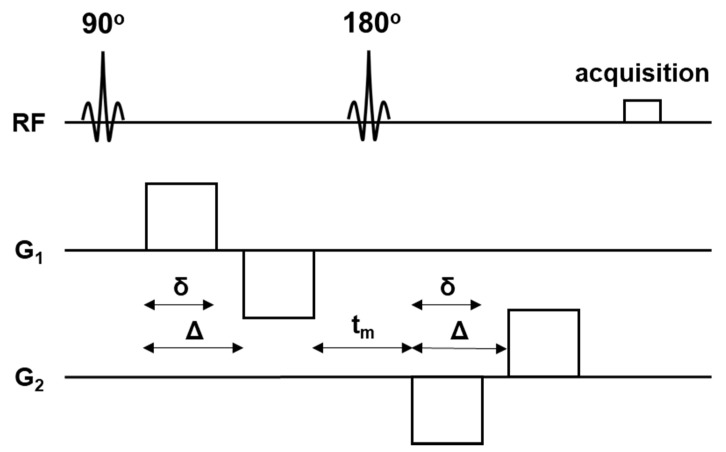
A schematic illustration of double diffusion encoding (DDE) pulse sequence design. The sequence consists of two diffusion gradient pairs in parallel and orthogonal directions (G_1_ and G_2_). RF = radiofrequency, δ = diffusion gradient pulse duration, Δ = diffusion gradient separation, t_m_ = mixing time.

MRI scans of the brain were performed using a 3T scanner (MAGNETOM Prisma, Siemens Healthcare, Erlangen, Germany) and a 64-channel head coil. DDE MRI was acquired using a prototype spin-echo echo-planar imaging (EPI) sequence using the following parameters: repetition time (TR)/echo time (TE) = 7000 ms/84 ms, diffusion gradient pulse duration (δ) = 12.2 ms, diffusion gradient separation (Δ) = 13.7 ms, b-value (b) = 0 and 800 s/mm^2^, the number of diffusion directions = 72, matrix = 128 × 128, field-of-view (FOV) = 192 × 192 mm^2^, voxel size = 1.5 × 1.5 × 4 mm^3^, GRAPPA acceleration factor = 2, acquisition time (TA) = 8:42 min, no slice gap, number of excitation (NEX) = 1. The sequence design is given in Figure 1. T1-weighted 3D magnetization-prepared rapid acquisition gradient echo (MPRAGE) sequence (TR/TE/ inversion time (TI) = 1900 ms/2.85 ms/900 ms, flip angle = 9°, voxel size = 0.9 × 0.9 × 0.9 mm^3^, NEX = 1) was acquired for anatomical information, tissue segmentation, spatial normalization of the reconstructed maps, and calculation of the brain volume. In addition, axial T2-weighted imaging (TR/TE = 4500 ms/87 ms) and fluid-attenuated inversion recovery (FLAIR) imaging (TR/TE/TI = 12,000 ms/115 ms/2800 ms) were also acquired to exclude gross abnormalities. A radiologist with 21 years of neuroimaging experience visually evaluated all images to exclude gross artifacts.

### 2.5. Data Processing

**Figure 2:**
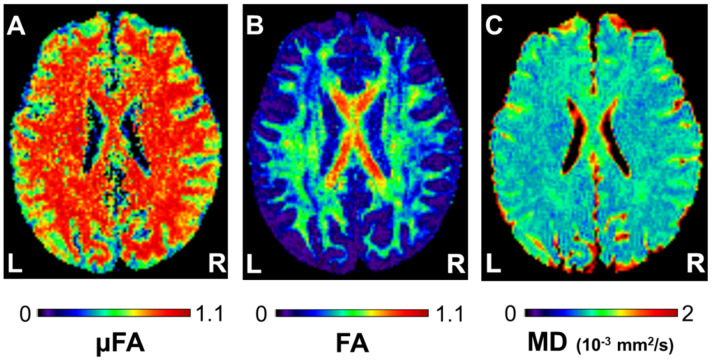
Example (**A**) microscopic fractional anisotropy (μFA), (**B**) fractional anisotropy (FA), and (**C**) mean diffusivity (MD) maps of a 23−year−old man. The values are given in the corresponding look−up tables. L and R indicate left and right, respectively.

From the DDE MRI data, we calculated μFA, FA, and MD maps using the following formulae:

(1) $$\mu \mathrm{FA}=\sqrt{\frac{\in }{\frac{2}{3}\in +\frac{1}{10}b^{2}{\left(\frac{1}{3}D\right)}^{2}}}$$

(2) $$\mathrm{FA}=\sqrt{\frac{{\left(\lambda_{1}-\lambda_{2}\right)}^{2}+{\left(\lambda_{2}-\lambda_{3}\right)}^{2}+{\left(\lambda_{3}-\lambda_{1}\right)}^{2}}{2\;(\lambda_{1}{}^{2}+\lambda_{2}{}^{2}+\lambda_{3}{}^{2})}}$$

(3) $$\mathrm{MD}=\frac{\lambda_{1}+\lambda_{2}+\lambda_{3}}{3}$$

where ε is the signal variation between the parallel and orthogonal diffusion gradient pairs; b is the b-value; D is the mean diffusivity of the diffusion tensor; and λ_1_, λ_2_, and λ_3_ are the eigenvalues of the diffusion tensor [15,22]. Calculations were done using a custom-built software that runs on MATLAB R2009b (The MathWorks, Natick, MA, USA). Example μFA, FA, and MD maps of a subject are given in Figure 2.

The reconstructed μFA, FA, and MD maps were first co-registered to the corresponding echo-planar images with no diffusion weighting (i.e., b = 0 s/mm^2^) to limit motion and eddy current-induced image distortions. Default parameters of Statistical Parametric Mapping 12 (SPM12) software (Wellcome Trust Centre for Neuroimaging, London, United Kingdom) were used. Next, these maps were co-registered between the two-time points and to the corresponding MPRAGE images. These steps were followed by spatial normalization of co-registered MPRAGE images to the standard Montreal Neurological Institute (MNI) space. The transformation parameters were then applied to the corresponding μFA, FA, and MD maps. The normalized μFA, FA, and MD maps were then smoothed with a Gaussian kernel of 3 × 3 × 8 mm full width at half maximum (FWHM). For the normalized MPRAGE images, a Gaussian kernel of 2 × 2 × 2 mm FWHM was applied.

The cerebrospinal fluid (CSF) of each participant was segmented from the corresponding normalized MPRAGE images (SPM12), and the CSF volumes were calculated for inclusion as a covariate in statistical analyses (MRICron version 1.0, Chris Rorden’s Neuropsychology Lab, University of South Carolina, Columbia, SC, USA) [23]. Mean normalized MPRAGE images were then generated by averaging the normalized MPRAGE images of all participants (SPM12). A brain parenchyma mask including only the gray and white matter voxels of the mean normalized MPRAGE images was then produced (SPM12). To limit partial volume averaging, the mask was further dilated for one voxel using ImageJ version 1.51 (National Institutes of Health, Bethesda, MD, USA) [24]. This mask was applied as an inclusion mask in all image analyses.

### 2.6. Statistical Analysis

A 2 × 2 mixed-design ANOVA was used to evaluate the training and time-related changes of μFA, FA, MD, and the gray and white matter volumes (SPM12). The type III sum of squares approach was employed to account for the variation in between-group cell frequencies [25]. Age, gender, years of education, and the CSF volume were taken as covariates. Statistical significance was set as uncorrected *p* < 0.001 for clusters containing at least 50 voxels for μFA, FA, and MD, and 10 voxels for the gray and white matter volumes. If any, the mean values of significant clusters were extracted (MRICron), and the changes between the initial and re-assessment were tested for correlation with changes in the assigned task performance, using partial correlation analyses. For this purpose, statistical significance was set as uncorrected *p* < 0.05. Age, sex, years of education, and the CSF volume were taken as covariates. Correlation tests were performed using Statistical Package for the Social Sciences (SPSS) software version 22 (IBM, New York, NY, USA).

## 3. Results

### 3.1. Cognitive Training-Induced Imaging Finding Changes

**Figure 3:**
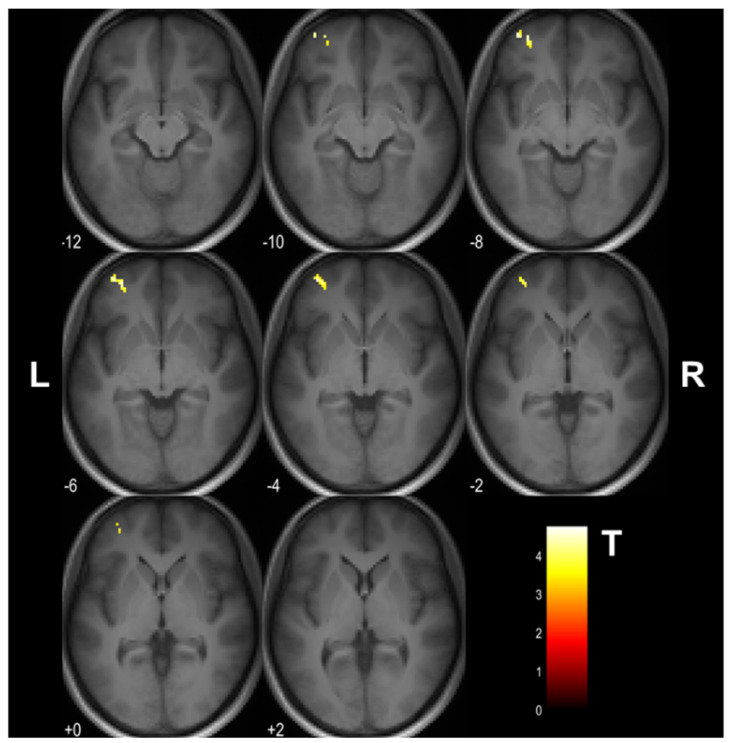
The cluster in the left middle frontal gyrus (MNI coordinates: X = −36, Y = 54, Z = −6) showing a decrease in μFA upon the 4 week cognitive training (uncorrected *p* < 0.001). The cluster is shown as an overlay on a 3D magnetization−prepared rapid acquisition gradient−echo (MPRAGE) template. The look−up table denotes T values. L and R indicate left and right, respectively.

**Figure 4:**
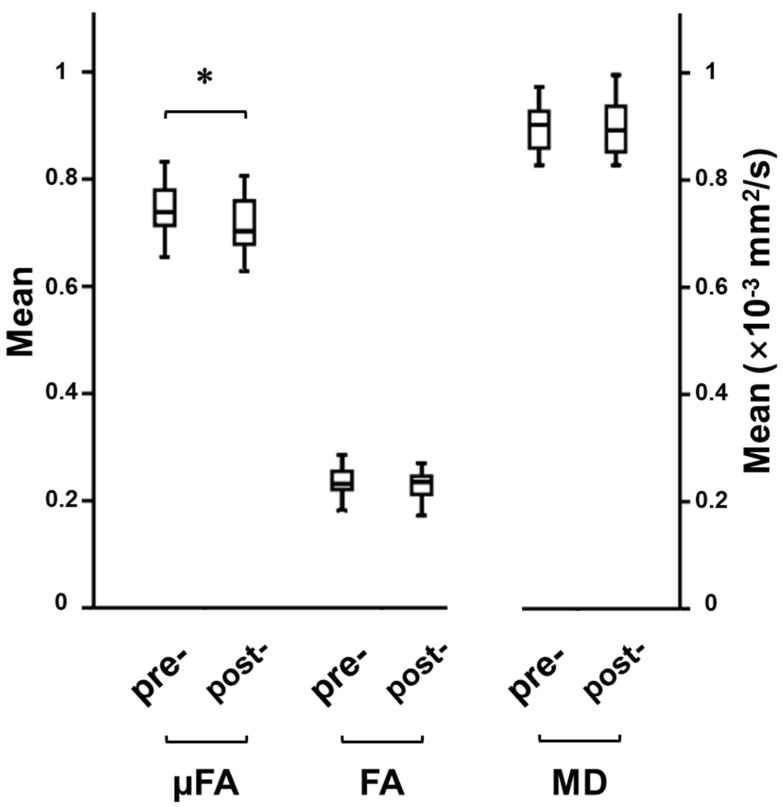
Box and whisker plots showing the median, interquartile range, minimum, and maximum for μFA, FA, and MD values of the cluster (left middle frontal gyrus) with significant training effect. * indicates statistical significance (uncorrected *p* < 0.001).

The training and time-related changes in μFA (F_(1,27)_ = 13.54, uncorrected *p* < 0.001) were observed on 2 × 2 mixed-design ANOVA. Post hoc analysis revealed that the training group had a μFA decrease in the left middle frontal gyrus (uncorrected *p* < 0.001; 53 voxels; MNI coordinates: X = −36, Y = 54, Z = −6) following the cognitive training (Figure 3). Forty-three of these voxels were located in the gray matter, and the rest white matter. The mean μFA value of this region and the corresponding mean FA and MD values are given in Figure 4. No voxel with significant μFA increase after the training was observed. There were also no significant time-related changes in μFA in the control group. Similarly, no significant time-related changes in FA, MD, and the gray and white matter volumes were observed in both groups.

### 3.2. Relationship between μFA and Task Performance

**Figure 5:**
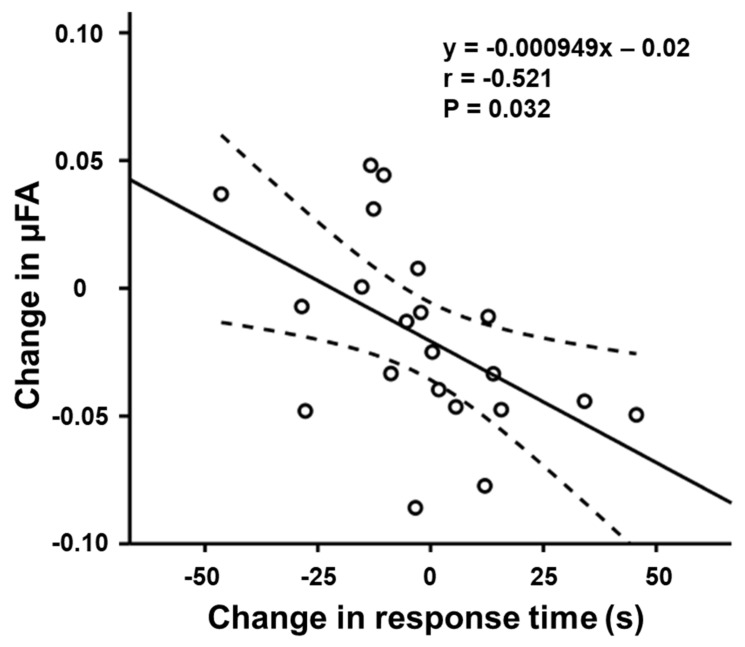
Scatterplots showing a significant negative correlation between the change in μFA of the left middle frontal gyrus and the changes in the response time for the orienting component of attention network test (r = −0.521, uncorrected *p* = 0.032). The straight and curved lines indicate the mean and 95% confidence interval.

The changes in mean task performance and their SD are summarized in Table 1. The results of correlation tests between the change in mean μFA in the left middle frontal gyrus and that of task performance are summarized in Table 2. There was a significant moderate negative correlation between the μFA change and the change in the RT for the orienting component of ANT in the training group (r = −0.521, uncorrected *p* = 0.032) (Figure 5).

## 4. Discussion

The results of this study highlight that μFA derived from DDE MRI could detect the cognitive training-induced neuroplastic changes. To our best knowledge, this is the first report evaluating cognitive training-induced microstructural brain changes by using μFA. The observations of training and time-related μFA change and its correlation with cognitive training task performance, but not FA and MD, are thought to suggest that μFA is more sensitive than FA and MD in evaluating microstructural brain changes.

μFA derived from DDE MRI is calculated from signal variation between the parallel (φ = 0°) and orthogonal (φ = 90°) diffusion gradient pairs, where φ is the angle between the gradient orientations [14]. On the other hand, FA and MD of DTI are calculated from the signal generated by a single diffusion gradient pair. The diffusion signal reflects an average of different diffusion profiles in each voxel, and encoding diffusion twice from different directions can quantify diffusion anisotropy without suffering from orientation dispersion; thus, μFA is considered more sensitive in characterizing microstructural diffusion anisotropy [14,16]. In principle, FA and µFA are identical in voxels containing coherently aligned anisotropic structures in which orientation dispersion approaches zero. However, FA decrease and preserved µFA can be observed in voxels of anisotropic structures with various orientations [26]. The superior sensitivity of μFA over FA has been reported in several preclinical studies. A computer simulation that involves anisotropic and isotropic tissue components has revealed higher sensitivity of μFA, over FA, in evaluating the changes in these tissue components [16]. An asparagus phantom study has observed high μFA and FA in intact asparagus (representing an anisotropic structure) and high μFA only in pureed asparagus (representing the microstructure with high orientation dispersion), suggestive of the higher sensitivity of μFA, or insensitivity of FA, to orientation dispersion [10]. To our knowledge, the superior sensitivity of μFA over MD in evaluating the microstructural changes has not been reported.

Neuroplasticity is the ability of the nervous system to respond to intrinsic or extrinsic stimuli by reorganizing its structure, function, and connections [27]. Cognitive training-induced neuroplastic changes have been reported in gray and white matter. A voxel-based study reported increased and decreased gray matter volume in several cortical regions, FA decrease in the prefrontal white matter regions, and MD increase in inferior parietal and cerebellar white matter regions, following a 6 week cognitive training [28]. Another voxel-based study observed increased gray matter volume in the right postcentral gyrus and increased functional connectivity between the right hippocampus and the left superior temporal gyrus in elderly subjects who underwent a 3 month cognitive training [3]. Studies that relate μFA to cognitive training are lacking, probably because μFA is a relatively new index. In the present study, μFA decreased in the left middle frontal gyrus upon cognitive training, reflecting neuroplastic changes such as a decrease in neuronal connectivity or the number of neurons, including axon terminals and dendritic branches. Such neuroplastic changes may be due to synaptic pruning. This neurological regulatory process facilitates changes in neural structure by removing redundant neurons and synaptic connections, leaving more efficient synaptic configurations [29]. The lack of volumetric changes implies that μFA changes occur earlier or are more sensitive than the volumetric changes in depicting neuroplasticity.

There were no significant training and time-related FA and MD changes in this study. This observation may reflect the lower sensitivity of DTI indices in unveiling trivial microstructural changes. Several previous studies have reported that DTI cannot represent multiple intravoxel orientations in regions of complex microarchitectures, such as neurites in the cortex and crossing fibers in the white matter [16,30]. Considering this limitation, the lack of change in FA and MD may be due to the dispersed orientation of neurites. The results of this study are consistent with a previous study that applied DTI and DDE MRI to evaluate the white matter microstructure in patients with Parkinson’s disease. In that study, a significant μFA decrease was observed in anterior corona radiata in the patients, but no FA change [31]. In this study, a negative correlation between the μFA change and the RT change for the orienting component of ANT was observed in the left middle frontal gyrus. The middle frontal gyrus is known to play an important role in the modulation of attention. Several functional MRI studies involving cued target detection tasks have reported a higher activation of reorienting of visuospatial attention in healthy volunteers and lower activation of attention in children with attention-deficit/hyperactivity disorder in bilateral middle frontal gyri [32,33]. Interestingly, the RT change exhibited both positive and negative values. From the observation of decrease in RT in both center- and spatial-cue conditions upon the training, the observed RT change is thought to indicate differential response to center- and spatial-cues within the training group. That is, some responded well when center-cues were presented, while the others performed better when spatial-cues were given. The lack of significant correlation of μFA with RT_alerting_ and RT_executive control_ may imply that the microstructural changes associated with improved orienting appear earlier than those for alerting and executive control. Another possibility would be the higher sensitivity of μFA to detect orienting-related microstructural changes. The lack of correlation of μFA to the DBT error rate may be due to insufficient statistical power, requiring further exploration.

Several limitations need to be addressed. First, being an exploratory study, correction for multiple comparisons was not performed in tests of correlation. The correlations would not thrive if correction was made. Nevertheless, we believe that the results of this study highlight the potential of DDE MRI in detecting anatomical areas involved in regulation of cognition. Second, the influence of handedness on the results was not evaluated. A functional MRI study has reported leftward lateralization among right-handers in visuospatial attention-related activations in middle frontal gyrus, and vice versa [34]. From the observation of lateralized microstructural brain changes, the influence of handedness on the results cannot be completely ruled out. Third, this study demonstrated the usefulness of μFA in revealing cognitive training-induced microstructural changes. We did not evaluate for how long these microstructural changes would last. This is beyond the scope of this study, but it would be worth to have a longitudinal follow-up. Finally, the brain’s microstructure can also be evaluated with other SDE MRIs, including diffusion kurtosis imaging and neurite orientation density and dispersion imaging [35,36]. This study is unable to determine whether DDE MRI is superior to these techniques.

## 5. Conclusions

This study evaluated the performance of μFA, FA, and MD in identifying microstructural brain changes associated with 4 week attention and working memory training. Cognitive training and time-related μFA changes were observed in the left middle frontal gyrus, a region that plays an important role in the modulation of attention. The changes in μFA correlated with changes in RT for the orienting component of ANT, suggesting that μFA can become a sensitive marker to evaluate cognitive training-induced improvement in attention.

## Figures and Tables

**Table 1:** Task performance in each group.

Items			Training Group (Mean ± SD)			Control Group (Mean ± SD)		
			Initial Assessment	Re-Assessment	Change	Initial Assessment	Re-Assessment	Change
ANT	RT_alerting_ (ms)		56.86 ± 27.50	48.95 ± 32.83	−7.90 ± 20.58	65.25 ± 14.91	61.00 ± 24.02	−4.25 ± 27.08
		*RT_no-cue_* *(ms)*	560.43 ± 39.08 **	529.19 ± 36.53 **	*−* *31.24 ± 24.82*	*550.25 ± 30.87*	*550.50 ± 28.56*	*0.25 ± 18.74*
		*RT_center-cue_* *(ms)*	503.57 ± 26.40 **	480.24 ± 28.20 **	*−* *23.33 ± 22.92*	*485.00 ± 25.88*	*489.50 ± 30.89*	*4.50 ± 17.64*
	RT_orienting_ (ms)		34.57 ± 21.27	32.90 ± 28.42	−1.67 ± 23.97	19.50 ± 13.27	29.75 ± 13.73	10.25 ± 17.46
		*RT_center-cue_* *(ms)*	503.57 ± 26.40 **	480.24 ± 28.20 **	−*23.33* ± *22.92*	*485.00 ± 25.88*	*489.50 ± 30.89*	*4.50 ± 17.64*
		*RT_spatial-cue_* *(ms)*	469.00 ± 33.14 **	447.33 ± 35.63 **	−*21.67* ± *21.82*	*465.50 ± 26.80*	*459.75 ± 30.44*	*−* *5.75 ± 11.73*
	RT_executive control_ (ms)		58.38 ± 23.16 **	36.92 ± 24.64 **	−21.46 ± 15.13	59.92 ± 29.16	54.54 ± 18.50	−5.37 ± 17.64
		*RT_incongruent_* *(ms)*	569.38 ± 42.87 **	522.51 ± 35.17 **	*−* *46.87 ± 21.86*	*560.17 ± 51.99*	*554.46 ± 43.09*	*−* *5.71 ± 21.20*
		*RT_congruent_* *(ms)*	511.00 ± 30.28 **	485.59 ± 28.91 **	*−* *25.41 ± 18.65*	*500.25 ± 26.50*	*499.92 ± 27.61*	*−* *0.33 ± 11.57*
DBT	Error rate (%)		37.38 ± 15.24 **	7.84 ± 7.89 **	−29.54 ± 10.24	43.75 ± 13.74 *	40.50 ± 12.92 *	−3.25 ± 3.43

Note: Items in italic are the RT for each cue and flanker condition. * and ** indicate statistical significance between the initial and re-assessment (* for *p* < 0.05 and ** for *p* < 0.01). ANT = attention network training, DBT = dual N-back training, RT = response time, SD = standard deviation.

**Table 2:** Relationship between the μFA changes in the left middle frontal gyrus cluster and task performance.

Item		Training Group		Control Group	
		Correlation Efficient (r)	Significance (*p*)	Correlation Efficient (r)	Significance (*p*)
ANT	RT_alerting_ (ms)	−0.163	0.533	0.314	0.686
	RT_orienting_(ms)	**−0.521**	**0.032**	−0.310	0.690
	RT_executive control_ (ms)	−0.093	0.721	0.199	0.801
DBT	Error rate (%)	0.147	0.573	−0.880	0.120

Note: The numbers in bold indicate statistical significance (*p* < 0.05). ANT = attention network training, DBT = dual N-back training, RT = response time.

## Data Availability

Not Applicable.
